# A Review on Autophagy in Orofacial Neuropathic Pain

**DOI:** 10.3390/cells11233842

**Published:** 2022-11-30

**Authors:** Mayank Shrivastava, Liang Ye

**Affiliations:** 1 School of Dentistry, University of Minnesota, Minneapolis, MN 55455, USA; 2 Department of Rehabilitation Medicine, University of Minnesota, Minneapolis, MN 55455, USA

**Keywords:** orofacial neuropathic pain, autophagy, nerve injury, somatosensory

## Abstract

Orofacial neuropathic pain indicates pain caused by a lesion or diseases of the somatosensory nervous system. It is challenging for the clinician to diagnose and manage orofacial neuropathic pain conditions due to the considerable variability between individual clinical presentations and a lack of understanding of the mechanisms underlying the etiology and pathogenesis. In the last few decades, researchers have developed diagnostic criteria, questionnaires, and clinical assessment methods for the diagnosis of orofacial neuropathic pain. Recently, researchers have observed the role of autophagy in neuronal dysfunction as well as in the modulation of neuropathic pain. On this basis, in the present review, we highlight the characteristics, classification, and clinical assessment of orofacial neuropathic pain. Additionally, we introduce autophagy and its potential role in the modulation of orofacial neuropathic pain, along with a brief overview of the pathogenesis, which in future may reveal new possible targets for treating this condition.

## 1. Introduction

Orofacial pain disorders include a broad range of clinically defined conditions, such as dental, musculoskeletal, neurovascular, and neuropathic pain. Patients suffering from orofacial pain disorders generally present to clinics with acute or chronic pain. Acute pain conditions are self-limiting and occur in response to tissue trauma or inflammation and are thus essential for survival. Chronic pain conditions on the other hand have a direct impact on patients’ functional and emotional components. It lasts or recurs for more than 3–6 months and can persist beyond normal healing time [1,2]. In some patients, chronic orofacial pain may persist when the peripheral lesions have been treated or even without peripheral abnormalities being found.

According to a study conducted by the US Centers for Disease Control, the prevalence of chronic pain ranges between 11% and 40%,with the point prevalence estimated to be 20.4% [3,4]. Moreover, the overall prevalence of orofacial pain varies from 5 to 57%, depending on the study population, location, and other factors, with women being affected more frequently in comparison to men [5].

In acute orofacial pain conditions, the peripheral mechanism predominates, though central sensitization may also contribute. While in chronic orofacial pain, both the peripheral and the central sensitization predominate; in addition, the development of new neural connections and brain alterations can be observed [3,6,7]. These changes are maintained not only by nociception, but also by psychosocial factors. The psychosocial factors associated with the perpetuation of chronic orofacial pain are anxiety, depression, post-traumatic stress, catastrophic thoughts, and poor social support. In addition, other contributing factors, such as genetics, hormones, sleep, age, and sex, may also contribute [8]. Overall, chronic orofacial pain conditions are associated with a dysfunction in the pain system that can also affect mood and severely worsen patients’ quality of life [9,10].

The classification of chronic orofacial pain falls into three main categories: nociceptive, neuropathic, and nociplastic or dysfunctional pain. The nociceptive pain commonly experienced in response to stimuli that have potential to damage tissues. It includes arthritis and muscle spasms and is commonly accompanied by anxiety and depression [3,11]. Orofacial neuropathic pain is defined as the pain caused by damage or diseases affecting the somatosensory nervous system. It is typically associated with positive and negative sensory abnormalities, such as allodynia, hyperalgesia, numbness, or both abnormalities as dysesthesia and paresthesia, and it is frequently accompanied by greater psychological distress and concomitant disability [12]. Unlike nociceptive pain and acute nerve injury, orofacial neuropathic pain is always maladaptive, with greater decrements in the quality of life [13]. Nociplastic pain, also known as dysfunctional pain, results from the abnormal processing of pain signals without any obvious signs of tissue damage or discrete pathology involving the somatosensory system. It includes non-specific back pain, some temporomandibular disorders (TMD), fibromyalgia, and irritable bowel syndrome. Indeed, there is mounting evidence that many pain conditions, such as headaches, cancer pain, and some orofacial pain conditions, have a mixed pain phenotype, meaning that they do not fit neatly into one category and that the mechanism underlying their etiology and pathogenesis is not well understood [14]. As a result, managing patients with a mixed phenotype of orofacial neuropathic pain is challenging for the clinicians.

Orofacial neuropathic pain conditions are difficult to manage, and the results are often unsatisfactory. It is observed that 15–25% of chronic pain is neuropathic pain. In a systematic review, the population prevalence of pain with neuropathic characteristics was noted as being between 6.9 and 10% [15,16]. The epidemiological research in this area is complex due to a lack of consensus on the valid definitions, diagnosis criteria, and the appropriate use of the screening tools used by previous studies.

Orofacial neuropathic pain is characterized by an altered sensation within a specific dermatome; it can be constant or intermittent and may be spontaneous or evoked. In some conditions, there is an obvious inciting event that produces the neuropathic pain, which can be peripheral or central in its origin (peripheral neuritis, post-herpetic neuralgia, and post-stroke pain), while pain can also be unrelated to disease or a traumatic event and may be diagnosed as primary trigeminal painful neuropathy (dysfunction of nerves in which the onset may be spontaneous or subsequent to various types of lesions of trauma) and persistent dentoalveolar pain type I [17]. Typically, in orofacial neuropathic pain the nervous system responds inappropriately to the damage via multiple mechanisms and their modulators, resulting in an imbalanced sensory system that misreads sensory inputs and can generate painful systems on its own. Additionally, there are different pain management approaches for orofacial neuropathic pain, despite the fact that patients do not experience adequate relief from the pain [18]. Due to this complex pathogenesis and lack of efficient treatment options, orofacial neuropathic pain is still an area of study.

There is growing evidence that autophagy dysfunction underlies neuropathic pain and that modifying autophagy can alleviate pain [19,20]. Autophagy is a cytoprotective process that replaces cell components in both constitutive and catabolic conditions. It is crucial for cell functions such as growth, inflammation, metabolism and aging. In general, autophagy promotes cell survival by allowing cells to adapt to stress conditions. Long-lived, dysfunctional, or excess proteins are degraded in this process to maintain normal tissue homeostasis [21,22].

Autophagy plays a vital role in synaptic maintenance and plasticity in CNS as well as for synaptic integrity and receptor turnover in the peripheral nervous system [23,24]. Mutations in autophagy-related genes have been linked to various disorders, including neuropathic pain [25]. In past decades, genetic studies have pointed to the involvement of autophagy in neurodegenerative diseases, cancers, and inflammatory and autoimmune diseases. However, autophagy activity cannot be precisely measured and little evidence has been generated to suggest that it is elevated or reduced in specific conditions. Experimental studies have shown that the activity of autophagy in the injured nerves is altered [26]. These studies showed that upregulated autophagy activities can directly alleviate neuropathic pain through the suppression of the pro-inflammatory cytokines which play a role in the formation of neuropathic pain [27,28,29,30]. Furthermore, the neural-specific depletion of the required autophagy genes causes axon degeneration and neuron death in experimental models. Its role in Schwann cell function during myelination and re-myelination has been also observed [31,32].

So far, there have been very few studies describing the role of autophagy in the dysfunction and modulation of neuropathic pain. In orofacial neuropathic pain, autophagy has not been explored well. Therefore, in this review we focus on orofacial neuropathic pain first, by presenting its classification, peripheral and central mechanisms, and assessment. Later, we discuss a brief view of autophagy, including how autophagy is executed and regulated at the molecular level and how it contributes to the development of neuropathic pain. Finally, we shed light on the therapeutic potential of autophagy, which may act as an emerging modulator of orofacial neuropathic pain, and we briefly discuss the clinical care of patients diagnosed with orofacial neuropathic pain.

## 2. Orofacial Neuropathic Pain

### 2.1. Characteristics and Types of Orofacial Neuropathic Pain

Orofacial neuropathic pain appears as a result of disease or lesion interferences involving the somatosensory system, which not only lead to increased pain sensitivity but also to loss of function. In general, patients with orofacial neuropathic pain complain of spontaneous ongoing pain which is occasionally dominated by intermittent shock-like pain paroxysms, either alone or in addition to the ongoing pain. The patients may also exhibit intermittent/recurrent painful episodes triggered by amplified pain responses after noxious or non-noxious stimuli [18].

The clinical presentation of orofacial neuropathic pain can be dependent on its origin and the insult that caused it. There are various nerve-damaging stimuli in the peripheral and CNS which can lead to neuropathic pain. The potential cause of orofacial neuropathic pain can be a trauma, infection, exposure to toxins, vascular/space lesion compression, or a metabolic disease, e.g., diabetic neuropathy, a neurodegenerative, autoimmune condition, a tumor, or a hereditary disease [17]. The various descriptors of orofacial neuropathic pain include burning, sharp, pricking, shooting, squeezing, tingling, and numbness, along with dysestheisa and paresthesia [18]. Typically, neuropathic pain can be divided into two general categories: those that are the consequences of peripheral lesions or disease, and those that are due to central lesions or disease. It can also occur as a condition of unknown etiology, or it can be idiopathic. The understanding of pain hypersensitivity and spontaneous pain in these idiopathic conditions is complex, and the underlying pathology still remains unclear. Before moving forward with the mechanism of orofacial neuropathic pain, we present here the different types of orofacial neuropathic pain, given that the differences in the pain phenotypes offers an opportunity to classify neuropathic pain clinically.

Neuropathic pain is generally classified in relation to the agent of insult and anatomic distribution [33]. There are various classifications of neuropathic pain reported in the literature. According to Woolf, a classification should be valid, reliable, and generalizable. However, the dynamic nature of the nociceptive system, particularly in abnormal conditions, may be an impediment to discovering such a universal classification [34]. It is difficult to meet the demands when orofacial neuropathic pain disorders are dominated by subjective symptoms and the associated clinical signs are few or nonexistent. Various scales and questionnaires have been developed in the literature to demonstrate discriminative features between neuropathic and non-neuropathic pain states. Recently, a grading system that classified neuropathic pain as possible, probable, and definite was updated [11]. The different levels were determined by the experts based on neurological history, pain distribution, the presence and location of sensory signs, and, finally, on a confirmatory test, neuropathic pain can be peripherally generated or centrally mediated. In some patients with post-herpetic neuralgia, central post-stroke pain, and compression by space-occupying lesions, the underlying cause of neuropathic pain is obvious, while for certain mixed conditions it is challenging for the clinician to delineate the boundaries of neuropathic and non-neuropathic pain.As a result, neuropathic pain is typically categorized according to an underlying disease. In the newly released ICD11 classification, neuropathic pain is first organized into peripheral and central neuropathic pain based on the location of the lesion or disease in the peripheral or central somatosensory nervous system [35]. Within each of these categories, the pain is classified into different neuropathic pain conditions based on the underlying disease.

**Figure 1:**
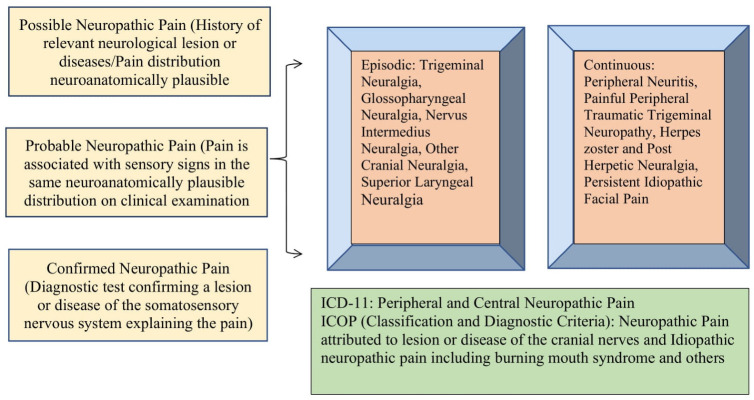
Types of orofacial neuropathic pain.

Moreover, based on the temporal features, orofacial neuropathic pain can be episodic or continuous. The episodic orofacial neuropathic pain included trigeminal neuralgia, glossopharyngeal neuralgia, and other cranial neuralgias, and the continuous pain included peripheral neuritis, peripheral painful trigeminal neuropathy, herpes zoster and post-herpetic neuralgia, and persistent idiopathic facial pain [17]. [Figure 1]. Recently, the ICOP (International Classification of Orofacial Pain) has provided a comprehensive description, along with the diagnostic criteria, of pain conditions affecting the orofacial region, including orofacial neuropathic pain [36]. The ICOP describes primary pain, which means pain that is not attributable to another disorder, as well as secondary pain, which is caused by another identified disorder, such as inflammation due to infection/autoimmune disease or trauma, sensitization of tissues, structural changes, and injury. In the ICOP, orofacial neuropathic pain defined as pain attributed to lesions or disease of the cranial nerves. The distribution of the trigeminal and glossopharyngeal nerve was used to categorize the neuropathic pain in this classification. It means pain that is restricted to the distribution area of one of the sensory cranial nerves (i.e., the trigeminal and glossopharyngeal) with a history of trauma or disease that is known to cause nerve injury. In this classification, trigeminal nerve pain is subcategorized based on the underlying neuropathic condition, such as trigeminal neuralgia, which includes classical, secondary, and idiopathic types, whereas other forms of trigeminal neuropathic pain other than trigeminal neuralgia are subcategorized based on the underlying causes, which include herpes zoster, post-herpetic neuralgia, post-traumatic trigeminal neuropathic pain with a subcategory of probable post-traumatic trigeminal neuropathic pain, idiopathic neuropathic pain, and other disorders. Additionally, idiopathic orofacial pain is explained as unilateral or bilateral intra-oral or facial pain in the distribution(s) of one or more branches of the trigeminal nerve(s) for which the etiology is unknown. It includes burning mouth syndrome, persistent idiopathic facial pain, and PDAP. However, for precise diagnostic criteria and full content, description references are included [37].

### 2.2. Pathophysiology of Orofacial Neuropathic Pain

The research on orofacial neuropathic pain has progressed well in the last few decades. The results from experimental pain models, quantitative sensory testing (QST), questionnaires, skin and nerve biopsies, and functional imaging have provided further insights into the underlying pathology of orofacial neuropathic pain. So far, the research has suggested that lesions or diseases of afferent pathways are related to the development of orofacial neuropathic pain. In fact, imbalances between the excitatory and the inhibitory somatosensory systems, changes in ion balance, and glial cell activation all contribute to the formation of neuropathic pain [18]. Moreover, the data indicate that not one but several mechanisms can lead to either the initiation or the progression of orofacial neuropathic pain. Importantly, many of these mechanisms do not depend on the causes of the diseases [33]. It is proposed that the same mechanisms can be observed in different orofacial pain conditions and that different mechanisms might be involved in a single patient and could lead to the same symptoms. This indicates the complexity of neuropathic pain. Additionally, the complexity also highlights the clinical importance of identifying the underlying pain mechanisms in individual patients; this would help the clinician in developing a mechanism-based treatment approach which would eventually provide more successful pain management. It is worth mentioning that pain mechanisms can be identified by assessing the patients’ individuals signs and symptoms. The data available so far can help us to understand the associations between the symptoms and the suggested underlying mechanisms and neurobiology of orofacial neuropathic pain.The potential mechanisms contributing to orofacial neuropathic pain are as follows.

The mechanism of orofacial neuropathic pain is distinct from the nociceptive pain system. In nociceptive pain, the nociceptors at the tissue damage site activate unmyelinated C fiber and thin delta fibers, which carry impulses to the higher centers of the brain. In orofacial neuropathic pain, the process is altered, and peripheral nerve damage or lesion is usually evident in both the injured and the neighboring intact (uninjured) nociceptive afferents [38,39,40].

In neuropathic states, the pain can be spontaneous or paroxysmal shooting that occurs in the absence of any external stimulus. This is usually caused by ectopic impulse generation within the nociceptive pathways and the peripheral and central sensitization.

For instance, in trigeminal nerve injury an inflammatory response is elicited and mediators such as prostaglandins (PG), pro-inflammatory cytokines, and chemokines are elevated, and all of these contribute to pain perception via direct stimulation of the primary sensory neurons [41]. Nerve injury also causes an increase in the expression of several receptor proteins, including transient receptor potential V1 (TRPV1).

TRPV1 is a physiological receptor that is located on specific subtypes of peripheral nociceptive terminals activated by noxious heat. In cases of nerve lesions or injury, TRPV1 is downregulated on the injured nerve fibers but upregulated on the uninjured C fibers. If the threshold of TRPV1 is lowered, the additional sensitization to heat by intracellular signal transduction might lead to spontaneous nerve activity induced by normal body temperature [42,43,44], which is frequently observed in patients with orofacial neuropathic pain. Clinically, patients with such underlying pain mechanisms can also be characterized by the presence of heat hyperalgesia in addition to ongoing burning pain. Similarly, aberrant nerve responses and increased expression of TRPM8 receptors have been also identified in response to cold [44]. This could explain why orofacial neuropathic pain patients experience more discomfort when their body temperature varies more or less than usual.

There is another mechanism related to orofacial neuropathic pain that has been documented in the literature and which until now has been observed in patients with burning mouth syndrome. One neurotrophic factor derived from glial cell lines, artemin, was discovered to have upregulated mRNA expression. This upregulated artemin signaling has been linked to TRPV1 hyper-expressions in tongue nociceptors via the p38 mitogen-activated protein kinase phosphorylation, resulting in tongue hypersensitivity [45]. Furthermore, following nerve injury, many monocyte-derived macrophages are known to infiltrate at the nerve injury site. These blood-borne macrophages accumulate particularly around injured axons, caused by monocyte chemoattractant protein-1 (MCP-1) signaling, which modulates the development of neuropathy [46]. Moreover, the infiltration and proliferation of macrophages releases insulin-like growth factor-1 (IGF-1) via TRPV2, and increases in TRPV4 expressions in the trigeminal ganglion (TG) have been observed to be involved in mechanical hypersensitivity in patients with neuropathic pain [47].

Additionally, in the peripheral and central nerve lesions, activation of immune cells (microglia) and migration of macrophages into the neurons and the ganglion were observed to increase the sensitivity of pain receptors to pro-inflammatory cytokines and tumor necrosis alpha (TNF-alpha), respectively. The activated microglia also release a number of immune modulators in the CNS which contributes to the maintenance of neuropathic pain [48]. It has also been reported that voltage-gated sodium channels and other ion channels undoubtedly experience alterations that affect the excitability of the nociceptive nerves [49]. Furthermore, the increasing levels of mRNA for the voltage-gated sodium channels seem to correlate with ectopic activity, and the increased expression of sodium channels in the lesioned and intact fibers might lower the action potential threshold until the ectopic activity takes place [50,51]. Similar changes in the central lesions were noticed within second-order nociceptive neurons, leading to central neuropathic pain. These inflammatory processes, as well as other changes within the context of peripheral nerve endings, contribute to peripheral sensitization [52].

Similarly, the central sensitization can develop as a result of ectopic activity in the primary afferent nerve fibers and the structural damage in the CNS itself may not be necessarily involved [53]. The continuous discharge of peripheral afferent fibers causes the upregulation of mediators such as TNF-alpha, excitatory neurotransmitters, nerve growth factor, and neuropeptides, which may result in post-synaptic changes in the second-order nociceptive neurons via the phosphorylation of the NMDA and AMPA receptors or the expression of the voltage-gated sodium channels [54]. These changes induce neuronal hyperexcitability, which allows mechanosensitive A beta and A delta afferent fibers with low thresholds to activate the second-order nociceptive neurons [55,56,57]. This may help in understanding why normal innocuous tactile stimuli, such as light brushing on the skin, become painful. Patients with central pain have also been reported to have similar mechanisms, and more detailed outlines are provided in other articles [7,18,33,53,58].

#### Neurobiology of Orofacial Neuropathic Pain

Peripheral neuropathic pain is caused by damage to the peripheral nervous system, and its continuation relies on maladaptive processes within the central nervous system which occur as a result of changes in the sensory, emotional, and other neural networks. After trigeminal nerve injury, discharges in the primary afferent neurons are conveyed to the trigeminal spinal sub-nucleus caudalis (Vc) and the upper cervical nerve (C1–C2) via TG neurons, resulting in severe persistent pain [59,60]. The hyper-activation of TG, Vc, and C1–C2, in association with non-neuronal glial cells and macrophages, are thought to be involved in neuronal hyperexcitability. It is known that non-neuronal glial cells and macrophages generate a variety of cytokines, neurotrophic factors, and tumor necrosis factors in TG, Vc, and C1–C2 [61,62]. These neuron–non-neuronal cell communications are also considered to be involved in the accumulation of more macrophages, which further accelerate the spreading of the neuronal activation, resulting in persistent pain. It was also noticed that the satellite cells, microglial cells, and accumulated macrophages released various cytokines and caused further enhancement of un-injured TG neurons, resulting in the spreading of the activation of TG neurons. Furthermore, in the Vc and C1–C2 regions, the microglia–astrocyte interaction is thought to be involved in the spreading of the excitability of the nociceptive neurons [60,63,64]. The combined effect of all the intercellular complex connections leads to the persistence of chronic orofacial neuropathic pain following nerve injury.

Furthermore, the noxious information from the brain stem neurons is further conveyed to the higher CNS areas. The major areas which receive noxious inputs from the Vc and C1-C2 regions are the ventral posteromedial thalamic nucleus (VPM), the medial thalamic nuclei (MT), and the parabrachial nucleus (PBN). These areas are known to be involved in the processing of orofacial pathological pain [65]. It has recently been shown that these ascending pathways are functionally modulated after trigeminal nerve injury [66]. Moreover, potent inhibitory neurons, such as descending pathways originating in the brainstem, contribute to the modulation of pain processing. A loss of inhibitory GABAergic transmission was observed; this leads to exacerbation of the orofacial neuropathic pain [67,68]. The lesions that affect the descending pathways, such as the monoaminergic and opioid systems, also lead to pain exacerbation via disinhibition. Studies have also reported that the alteration of the thalamocortical pathway, as well as the hypoactivity of the thalamus, is associated with central neuropathic pain.Additionally, cognitive affective factors such as anxiety, depression, and cognitive reappraisal are critical to the shaping of the experience of chronic orofacial neuropathic pain [53]. However, it looks like there are multiple mechanisms involved in genesis of neuropathic pain and we still do not have an appropriate strategy to diagnose and treat this orofacial pain because the detailed mechanisms underlying the pathological pain associated with trigeminal nerve injury are not fully understood.

### 2.3. Assessment of Orofacial Neuropathic Pain

The orofacial complex comprises many hard and soft tissue structures, such as the sinuses, masticatory structures, teeth, gingiva, and others, which are innervated by the trigeminal system. Given this complexity of structures, it is hard for the clinician to diagnose orofacial neuropathic pain. It is important to note that neuropathic pain in the orofacial area presents different challenges in comparison the neuropathic pain in other regions of the body. According to the International Headache Society, the orofacial region’s anatomic limits and related medical demarcations are factors contributing to the issue [36,37]. Moreover, neuropathic pain symptoms can mimic odontogenic tooth ache and lead to incorrect diagnosis. For example, trigeminal neuralgia involving the mandibular nerve is the most common, and paroxysmal pain is often felt in the tooth. It can lead to the diagnosis of endodontic pain and unnecessary endodontic treatment [69]. Another reason is that when nerves in the orofacial regions are injured, neuropathic pain induces ectopic or extraterritorial pain (area outside the innervation territory). In addition, referred pain from adjacent structures can easily lead to the misdiagnosis of dental pain, which in turns results in needless irreversible dental procedures, such as pulpectomy or extraction [70]. Therefore, accurate assessment of the orofacial neuropathic pain is critical to avoid unnecessary dental treatment [71].

The diagnostic criteria for a range of orofacial neuropathic pain conditions have already been described in the ICOP [36,37]. A clinician must have a basic understanding of the diagnostic criteria in order to be likely to arrive at a specific orofacial neuropathic pain diagnosis. There is the possibility that patients may have multiple pain diagnoses, which affects their prognosis. Therefore, it is critical to conduct a detailed interview of the patients. The descriptive history should include the likely cause, location, quality, duration, frequency, and other pain descriptors, as well as the psychological, social, and medical history. In some cases, the history of third molar extractions, implant placement, root canal therapy, orthognathic surgery, facial fractures, viral infection, and stroke should be documented in order to reach a specific diagnosis of post-traumatic trigeminal neuropathy, post-herpetic neuralgia, and post-stroke pain [59]. This interview should be followed by a thorough clinical examination of the orofacial structures, including a dental, TMD, cervical, and cranial nerve examination.

It is also important to have valid diagnostic tools that differentiate between neuropathic pain and nociceptive pain. Patients with neuropathic pain often have areas of abnormal sensation and hypersensitivity in the affected area, which can be adjacent to or combined with other areas of sensory deficit. The signs are hypoaesthesia (reduced sensation of non-painful stimuli), hypoalgesia (reduced sensation of painful stimuli), paresthesia (skin crawling or tingling sensation), hyperalgesia (increased pain sensitivity to a nociceptive stimulus/pain from normally non-painful cold/heat/mechanical stimuli), allodynia (pain from normally non-painful light moving stimuli), spontaneous ongoing pain, and evoked shooting, electric shock-like situations. Other features, such as summation, which is the progressive worsening of pain evoked by slow repetitive stimulation with mildly noxious stimuli, can be also assessed.

For the assessment of a neuropathic region, a sensory examination may be performed. It includes mechanosensory tests that are responsive to light touch and temperature and painful and vibratory stimuli. These findings can be assessed by the clinician with gentle mechanical stimuli, such as strokes with a painters brush, cotton swab and gauze, pin pricks, stiff von Frey hair, and a cold/hot glass of water. The associated signs inducing autonomic changes must be assessed. Reflex assessment and quantitative sensory testing may be used aa additional methods for assessment. These sensory examinations would help the clinician to diagnose orofacial neuropathic pain and distinguish it from nociceptive pain. It is accepted that the assessment should be carried out in the area of pain, with the contralateral area as a control. Moreover, in orofacial neuropathic pain the distinction between the primary and secondary areas, which correspond to the tissue supplied by the damaged nerves and the area outside the innervation territory, should be assessed, respectively, as mechanical hypersensitivity often expands into the secondary area [18].

There are validated diagnostic screening tools available that can be used to differentiate between neuropathic pain and other pain dimensions. The McGill Pain Questionnaire, the Leeds Assessment of Neuropathic Symptoms and Signs (LANSS), the self-reported LANSS, the Neuropathic Pain Questionnaire (NPQ), the Douleur Neruropathique en 4 (DN4) questions, the PainDETECT questionnaire, and ID Pain are among them [17,72]. Previous studies reported that the DN4 and the NPQ were the most suitable for clinical use and might be used to assess the efficacy of the treatment response. Their sensitivity and specificity for chronic pain in the trigeminal system, however, are poor [73]. Other medical screening tests included hematology, glucose, creatinine, liver function test, renal function test, thyroid function, vitamin B12, serum protein immunoelectrophoresis, genetic testing, etc. Imaging techniques may also be required to exclude the local cause of nerve damage; usually, plane films and cone beam computed tomography (CBCT) and magnetic resonance imaging (MRI) and magnetic resonance angiography (MRA) of brain imaging are needed if a central lesion is suspected.

## 3. Autophagy

Autophagy is an intracellular degradation process that is essential for balancing energy sources during development. This process also aids in the survival of cells in a stressful environment. Different autophagy responses, such as macro-autophagy, micro-autophagy, and chaperone-mediated autophagy, have been identified through molecular research. These responses all promote the proteolytic degradation of cytosolic components at the lysosome [74].

The primary regulated form of autophagy that responds to physiological and environmental signals is known as macro-autophagy. In this process, a portion of cytoplasm is engulfed by a thin membrane cistern known as the isolation membrane or phagophore, resulting in the formation of a double-membrane organelle called the autophagosome. Upon the fusion of the outer autophagosomal and the lysosomal membrane, the lysosomal enzymes degrade the inner autophagososmal membrane and the enclosed material. It is considered a non-selective process which is now known to break down specific contents such as damaged mitochondria (mitophagy), lysophagy (ruptured lysosomes), xenophagy (intracellular microbes), etc. Autophagy flux refers to the entire autophagy process, which represents the dynamic process of the autophagy from cargo sequestration to its degradation [22,25,75].

The macro-autophagy process involves the orchestrated action of multiple complex proteins encoded by autophagy-related genes (ATG) which were identified in yeast. These ATG proteins are divided into four subgroups: Atg1/unc-51-like kinase (ULK) complex, which regulates the initiation of autophagy; two ubiquitin-like proteins, Atg12 and the Atg8/microtubule-associated protein light chain 3 (LC3) conjugation systems, which aid in the elongation of the autophagy membrane; the class III phosphatidylinositol 3-kinase (PI3K)/Vps34 complex I involved at the early stage of the autophagosome membrane formation; and two trans brand proteins, Atg9/mAtg9 and VMP1, which contribute to the delivery of the membrane in forming an autophagosomes [25,76].

In general, the two main phases in the autophagy process are the induction of the autophagosome and the fusion of the autophagosome with lysosome. In response to insulting conditions, upstream factors, mainly Beclin-1, are activated, resulting in the formation of a phagophore. In the latter phases, the autophagy-related genes (ATGs; ATG-5,-12,-16L, and -7), along with other effectors, such as P62 and microtubule-associated protein 1A/1B-light chain 3 (LC3)-II, accelerate the formation of the autophagosomes. Subsequently, the fusion of the autophagosomes with lysosomes contributes to the formation of autophagolysosomes, which in turn recycles or effluxes the contents out of the host cell [22,77].

In micro-autophagy, the cytosolic components, such as proteins and organelles, are directly taken up by the lysosome itself via invagination of the lysosomal membrane. Macro- and micro-autophagy are capable of engulfing the large structures through both selective and non-selective methods [76].

Chaperone-mediated autophagy can assist the host cells in adapting to the insulting conditions by activating heat shock protein-70 (HSP70) and lysosome-associated membrane protein type-2A (LAMP2A) [22,75]. In this process, cytosolic chaperone heat shock cognate 70 kDa protein (HSc70) was found to recognize and bind a specific KFERQ-like pentapeptide motif, resulting in the selective delivery of single protein substrate to lysosomes. The chaperone–substrate complex binds to the cytosolic tail of LAMP-2A, causing it to assemble into a protein complex and then into a multimeric complex that mediates substrate translocation. After complete unfolding, the substrate enters the lysosomal matrix for degradation with the aid of luminal chaperones [78].

### 3.1. Autophagy in Orofacial Neuropathic Pain Formation

Autophagy is generally regulated by a variety of stresses, including reactive oxygen species (ROS), mitotoxicity, inflammation, and endoplasmic reticulum (ER) stress. The influence of these stresses on autophagy has been studied in the literature on neuropathic pain models [25]. From these studies, it is observed that ROS contribute to peripheral and central sensitization following nerve injury, resulting in neuropathic pain. ROS have been found to be unregulated in microglia, astrocytes, and spinal neurons in studies. Furthermore, blocking the mitochondrial electron chain reduces hyperalgesia in a range of neuropathic pain models [79,80]. Although neuropathic pain causes baseline activation of autophagy in response to ROS formation, a significant degree of ROS-mediated oxidative stress also aids in the replacement of damaged components with new healthy ones [81,82]. Overall, ROS have been shown to both positively and negatively regulate autophagy depending on the levels and context. Similarly, Mitochondrial dysfunction has been found to be a major mechanism underlying the neuronal dysfunction associated with peripheral neuropathies; however, its role in orofacial neuropathic pain has not been explored. Typically, mitochondrial dysfunction in neurons leads to disturbed neurotransmission, and defective autophagy signaling is known to be associated with neuropathic pain [28,83,84]. Hence, the identification of drugs which sustain the mitochondrial function and health could aid the search in finding a better therapeutic strategy, which might open new prospects in the treatment of orofacial neuropathic pain. Another stressor, ER stress, means the accumulation of unfolded proteins in cells. This is also considered to be involved in the induction and maintenance of neuropathic pain. However, more studies are needed to investigate the relationship of different stressors in orofacial neuropathic pain.

**Figure 2:**
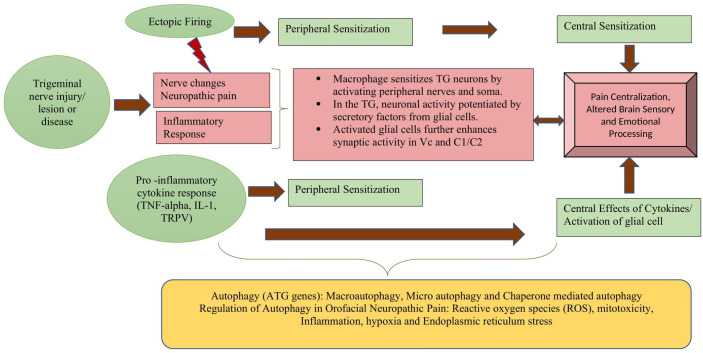
Role of Autophagy in pathogenesis of Neuropathic pain.

There are studies that show the dysfunction of autophagy in animal models of neuropathic pain. The data showed autophagy disruption in the spinal cord and an increase in the level of microtubule-associated protein 1 light chain 3 (LC3)-II, LC3-binding protein p62 and LC3, and Beclin 1. According to the literature, constitutive autophagy has a significant neuroprotective effect, while the dysregulation of autophagy can increase neuronal hyperexcitability and predispose to the development of neuropathic pain. It has also been observed that downregulation of the Atgs in neurons, as well as the accumulation of endoplasmic reticulum in the neuronal axons, may result in inefficient calcium-dependent excitatory nerve transmission. It can be assumed that after trigeminal nerve injury the stimulation of autophagy-related effectors in Schwann cells can compensate for the injury by engulfing the myelin debris associated with neuropathic pain. Similarly, animals studies have shown that in knockout Atg7 mice and Atg5-deficient mice, cellular death occurred in different brain regions, indicating protective role for brain parenchyma as well as the intracellular accumulation of P62-tagged (molecule that sensitized neurons) cytoplasmic inclusion bodies noticed in motor and sensory deficits with behavioral abnormalities. These findings imply that autophagy plays a crucial role in neuropathic pain and can be of particular importance for further research; however, its role in orofacial neuropathic pain has not been established yet [22,84,85,86]. [Figure 2].

Nearly all of the studies mentioned above that revealed an increase and decrease in autophagy are expressed in neuropathic animal models. Despite the contribution of ROS to neuropathic pain, which might upregulate autophagy, until now it is unclear what mechanisms cause the dysfunction of autophagy following neuropathic pain. Further investigations are needed to identify the status of autophagy in different neuropathic pain models specifically for orofacial neuropathic pain.

### 3.2. Therapeutic Potential of Autophagy in the Management of Orofacial Neuropathic Pain

According to the literature, it is clear to some degree that dysregulation of autophagy may participate in the process of neuropathic pain. Studies have documented that autophagy modulation in the DRG neurons can relive peripheral nerve pain and promote neuronal healing [25,30]. A recent study observed that the synthesis of nerve growth factor substantially accelerates debris clearance and enhances axonal regeneration through the stimulation of autophagy [86]. Correspondingly, in order to reduce the pain behavior in animal models of neuropathic pain, researchers have used drugs, inhibitors that affect autophagy. However, in orofacial pain models the modulation of pain via stimulating autophagy is yet to be explored. In this section, we are discussing the major agents used to alleviate neuropathic pain by controlling the autophagy.

#### 3.2.1. Rapamycin

The mTOR inhibitor rapamycin is commonly used in vivo to promote autophagy. It has been observed that rapamycin is effective in the management of neuropathic pain. The neuroprotective effects of rapamycin are typically mediated by the restoration of the autophagy–lysosomal pathway. It is presumed from the studies that the intrathecal injection of rapamycin can reduce thermal hyperalgesia and mechanical allodynia in rodents. Some studies reported that rapamycin can have anti-inflammatory effects, facilitate nerve regeneration, and prevent pain chronification [87]. The results of certain studies indicated a favorable link between the expression of neurofilament (NF)-200 and myelin basic protein (MBP), which participate in major axonal projections and myelination, respectively, [88] and an autophagy induction by rapamycin. In parallel with these findings, autophagy exerts neuroprotective effects on peripheral nerve and motor function recovery [89]. In addition to autophagy, rapamycin is also known to affect other cellular functions, such as protein synthesis, cell proliferation, and immune responses. However, the effectiveness of rapamycin so far has been explored in peripheral nerve injury or lesions, not in central nerve injury-induced neuropathic pain, which can be an area of future research.

#### 3.2.2. RNA Agents

RNA agents, including microRNA and siRNA, have also been used in studies to regulate pain processing in a wide range of experimental models. MicroRNAs (miRNAs) are non-coding single-stranded RNA of 19–24 nucleotides that mediate post-transcriptional gene silencing to regulate gene expressions and promote microglia activation [90,91,92]. It is documented that MiR-145 injection at the DRG has been shown to alleviate allodynia and thermal hyperalgesia [93]. Another study reported that miR-183 can suppress neuropathic pain and AMPA receptors by inhibiting the mTOR/vascular endothelial growth factor (VEGF) pathway in rodents [94]. It has been demonstrated that miRNAs can be used as potential targets for therapeutic interventions in patients with neuropathic pain.

Other major agents, such as melatonin, have been shown to reduce neuronal excitability in a subpopulation of DRG neurons. They also improve neurological deficits in rodents by increasing the autophagy pathway in peripheral nerves and the DRG. Similarly, progranulin, which is expressed in neurons and microglia, can improve autophagy and pain behavior in the injured nerves [95]. Another agent, chloroquine, which is an autophagy inhibitor, acts by inhibiting lysosomal proteases and autophagosome lysosomal fusion events and has been shown to be involved in pain processing [96].

**Figure 3:**
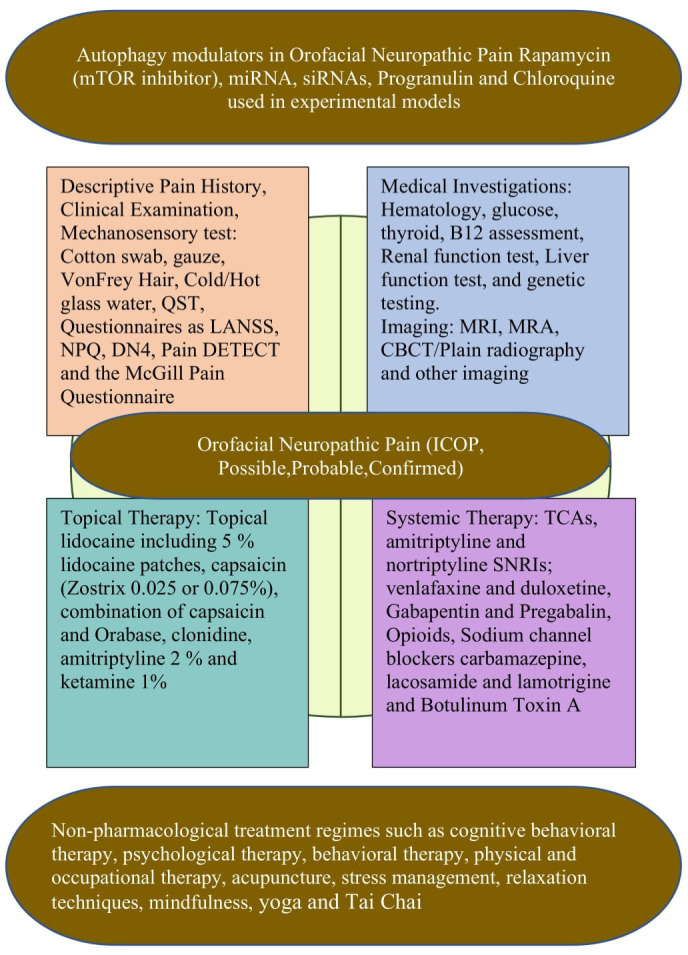
Summary of therapeutic potential of autophagy and clinical management of orofacial neuropathic pain.

However, in all the above studies different experimental models have used. The role of autophagy has not been explored in orofacial neuropathic pain models. The progress in assessing the role of autophagy in human diseases and their treatment relies heavily on the development of methods for monitoring autophagy activity in humans. More studies are needed to understand and assess the role of autophagy in the modulation of orofacial neuropathic pain in experimental models and humans (Figure 3).

### 3.3. Overview on the Management of Orofacial Neuropathic Pain

The management of orofacial neuropathic pain is challenging as many patients do not experience adequate relief in pain, as determined from the clinical trial outcomes. Ineffective interventions continue to be hampered by the lack of an understanding of the physiological basis of orofacial neuropathic pain. This ineffectiveness could also be due to the heterogeneity of neuropathic pain mechanisms as well as the frequently coexisting psychological and emotional aspects of chronic pain. In the treatment plan, patients should first be educated, including by information on orofacial neuropathic pain and treatment options. When beginning symptomatic treatment, potential drug side effects, drug interactions, and the importance of being compliant with the medications should all be addressed. Unrealistic patient expectations should be avoided, and patients should be informed about realistic treatment goals which might result in either partial or complete pain relief. In addition to pain, sleep disturbances, and coexisting anxiety depression which might hinder pain management should be identified and targeted for specific treatment. Hence, an interdisciplinary therapeutic approach, including pharmacological and non-pharmacological treatment regimes, such as cognitive behavioral therapy, psychological therapy, behavioral therapy, physical and occupational therapy, acupuncture, stress management, relaxation techniques, mindfulness, yoga, and Tai Chi should be taken into account, based on the previous literature [17,18].

In pharmacological approaches, the topical medications that are applied orally to the injured or sensitive site and are used in conjunction with a neurosensory stent have proven to provide adequate comfort to the patients. Locally acting drugs have an advantage over centrally acting drugs in that they are less likely to induce systemic side effects; they interact less with other medications and can provide faster relief. Moreover, the discontinuation of systemic drugs can result in side effects which are less common with topical medications. In general, topical medications are used to treat peripheral targets such as post-traumatic trigeminal neuropathy, which involves peripheral sensitization.

Topical lidocaine has been shown in studies to desensitize the peripheral ectopic generator in patients with trigeminal neuralgia and post-herpetic neuralgia. Typically, 5% lidocaine patches provide excellent relief from allodynia in patients with slight sensory loss, but not in those with profound sensory loss [97]. Another topical medication, capsaicin (Zostrix 0.025–0.075%), was found to be effective in patients complaining of a burning sensation, which acts by depleting the substance P in C-fiber primary afferents, thereby reducing the peripheral noxious inputs. A combination of capsaicin and Orabase (a paste containing 16.7% gelatin, 16.7% pectin, and carboxymethylcellulose sodium 16.7%) was also found to be effective in the treatment of orofacial neuropathic pain [98]. When sympathetic involvement is suspected, clonidine can be added to the formulation, which suppresses the norepinephrine release from the sympathetic terminals [99]. In addition, clinicians have applied a formulation containing 2% amitriptyline and 1% ketamine and noted an analgesic effect in orofacial neuropathic pain [100].

For severe pain, a combination of systemic and topical medications is often required. According to the Neuropathic Pain Special Interest Group (NeuPSIG) of the International Association for the Study of Pain (IASP), the drugs with a moderate to high quality of evidence and strong recommendations included tricyclic antidepressants (TCA), gabapentin, pregabalin, and serotonin noradrenaline reuptake inhibitors (SNRI: duloxetine and venlafaxine). These are first-line medications for orofacial neuropathic pain. TCAs and SNRI inhibit the pre-synaptic re-uptake of serotonin and noradrenaline, and their analgesic effects are thought to be due to the activation of a descending pathway at the spinal and supra spinal sites, though peripheral mechanisms are also suggested to be involved, while the gabapentin and pregablin analgesic actions are due to the inhibition of the voltage-gated calcium channels and the reduced activity-dependent calcium signaling; thereby, they inhibit the excitatory transmitter release and reduce neuronal hyperexcitability. Other actions, such as an effect on glia cells and an expression of pro-inflammatory cytokines, may also be involved. These recommendations were published in the literature based on the systematic review and meta-analysis of the published and unpublished randomized controlled double-blind trials [17,18,33,59,70,101]. Capsaicin 8% (acts on TRPV1), lidocaine patches (blocks voltage-gated sodium channels), and botulinum toxin injection A for peripheral neuropathic pain only received a weak recommendation. The precise mechanism of the botulinum toxin injection in the periphery is not known, but it is suggested to be involved in reducing inflammation, inhibiting neuropeptide and neurotransmitter release from primary afferents, reducing sodium and TRPV1 activity, or the central effects via retrograde transport. There was inconclusive evidence for the sodium channel blockers carbamazepine, lacosamide, and lamotrigine, but these medications are effective in the subgroups of orofacial neuropathic pain, such as trigeminal neuralgia. Opioid analgesics are agonists at the presynaptic and postsynaptic opioid receptors. Efficacy has been reported in several randomized, controlled trials in different peripheral and central neuropathic pain disorders. Tramadol inhibits serotonin and norepinephrine reuptake and can therefore interact with serotoninergic drugs (selective norepinephrine reuptake inhibitors and selective serotonin reuptake inhibitors), causing a serotonin syndrome. Opioids have comparable analgesic efficacy in TCA but long-term side effects, physical dependency, misuse, or abuse limit its use in patients with non-cancer neuropathic pain [18,33,59].

Despite the evidence for the efficacy of drugs with different mechanisms, many patients noticed side effects and did not obtain adequate pain relief at a tolerated dose. In such cases, if one drug is only partially effective a combination therapy may be used. In refractory cases, spinal drug administration or neuromodulation may be considered; however, there is little evidence observed from the randomized clinical trials. With similar low evidence, transcutaneous electrical stimulation is commonly used as a non-invasive interventional therapy in patients with neuropathic pain. Neural blockade, sympathetic nerve block, and epidural blocks are also recommended for patients with post-herpetic neuralgia, other subgroups of orofacial neuropathic pain, and complex regional pain syndrome. Unfortunately, orofacial neuropathic pain is considered refractory to most available treatments, likely because treatment is offered after pain has already developed. It is possible that irreversible neuronal damage has already occurred, limiting the therapeutic potential. Therefore, early preventive strategies to mitigate orofacial neuropathic pain may be needed.

## 4. Conclusions

This review outlined various types of orofacial neuropathic pain conditions as well as diverse clinical features, demonstrating that there are multiple factors other than nerve lesions that contribute to chronic pain manifestation. Despite ongoing research on the mechanisms underlying the neuropathic pain, the findings in this review show basic pathology associated with trigeminal nerve injury and nerve lesions. Additional screening methods for orofacial neuropathic pain are discussed. These methods will assist clinicians in making a proper diagnosis and comprehending the disorder. The present review also describes the crucial area of research in neuropathic pain as autophagy. We discussed new insights into the role of autophagy in orofacial neuropathic pain, including the types and how autophagy is regulated by diverse stresses in neuropathic formation. Though previous studies have investigated autophagy in neuropathic pain, more credible data describing the autophagy dysfunction and its role in modulation of neuropathic pain need to be explored. So far, the role of autophagy in the modulation of neuropathic pain is studied in experimental models but human studies are needed. We also provide drug inhibitors which modulate autophagy and are capable of preventing the neuropathic pain and its chronification in experimental models. Future studies are needed to mitigate the issues of the unclear cellular and molecular mechanisms which induce the dysfunction of autophagy. Research needs to be undertaken to introduce more effective management options for orofacial neuropathic pain patients. The development and validation of biomarkers might help to identify the patients who are at more risk of orofacial neuropathic pain and who therefore might benefit from pharmacological or non-pharmacological strategies. This paper raises the prospects that soon we will be able to target the orofacial neuropathic pain conditions with new approaches, which will enhance the patient prognosis.
